# Planar cell polarity genes *Celsr1* and *Vangl2* are necessary for kidney growth, differentiation, and rostrocaudal patterning

**DOI:** 10.1016/j.kint.2016.07.011

**Published:** 2016-12

**Authors:** Hortensja Ł. Brzóska, Angela M. d’Esposito, Maria Kolatsi-Joannou, Vishal Patel, Peter Igarashi, Yunping Lei, Richard H. Finnell, Mark F. Lythgoe, Adrian S. Woolf, Eugenia Papakrivopoulou, David A. Long

**Affiliations:** 1 Developmental Biology and Cancer Programme, UCL Great Ormond Street Institute of Child Health, London, UK; 2 Centre for Advanced Biomedical Imaging, Division of Medicine, University College London, London, UK; 3 Department of Internal Medicine, University of Texas Southwestern School of Medicine, Dallas, Texas, USA; 4 Department of Medicine, University of Minnesota, Minneapolis, Minnesota, USA; 5 Dell Pediatric Research Institute, Department of Nutritional Sciences, The University of Texas at Austin, Austin, Texas, USA; 6 Institute of Human Development, Faculty of Biology, Medicine and Health, University of Manchester, Manchester, UK

**Keywords:** kidney development, planar cell polarity, renal malformations, rostrocaudal patterning, ureteric branching

## Abstract

The mammalian kidney contains nephrons comprising glomeruli and tubules joined to ureteric bud–derived collecting ducts. It has a characteristic bean-like shape, with near-complete rostrocaudal symmetry around the hilum. Here we show that *Celsr1*, a planar cell polarity (PCP) gene implicated in neural tube morphogenesis, is required for ureteric tree growth in early development and later in gestation prevents tubule overgrowth. We also found an interaction between *Celsr1* and *Vangl2* (another PCP gene) in ureteric tree growth, most marked in the caudal compartment of the kidneys from compound heterozygous mutant mice with a stunted rump. Furthermore, these genes together are required for the maturation of glomeruli. Interestingly, we demonstrated patients with *CELSR1* mutations and spina bifida can have significant renal malformations. Thus, PCP genes are important in mammalian kidney development and have an unexpected role in rostrocaudal patterning during organogenesis.

The mammalian kidney contains nephrons, comprising glomeruli and tubules, joined to ureteric bud–derived collecting ducts. The mature kidney has a bean-like shape, with near-complete rostrocaudal (top to bottom) symmetry around the organ’s hilum. The growth of the embryonic metanephros is divided into 2 major phases.^1^, ^2^ The first growth phase is dominated by serial ureteric bud branching to form a tree of collecting ducts and bud tips. The first branch point of the bud within the just-formed metanephros defines the kidney’s rostral and caudal domains, and a mixture of predominantly dichotomous branching and some trichotomous branching establishes the organ’s bean shape.^3^ The second growth phase is dominated by nephrons that differentiate into podocytes, proximal tubules, and loops of Henle, together with the continued branching of the ureteric tree in the outer cortex. In addition, the collecting ducts in the medulla elongate and mature to form the papilla, which projects into the renal pelvis.

Planar cell polarity (PCP) describes the orientation of structures within an epithelial sheet. PCP is involved in processes that occur in normal development and differentiation including directional cell movement, polarized cell division, ciliary orientation, and convergent extension.^4^ PCP has been implicated in neural tube closure, and mutations of PCP genes cause a spectrum of neural defects in mice,^5^ with PCP gene variants associated with subsets of humans with similar anomalies.^6^, ^7^, ^8^ Two of these genes are *Van Gogh-like 2* (*Vangl2*) and *Cadherin EGF LAG seven-pass G-type receptor 1* (*Celsr1*). Missense mutations in *Vangl2* and *Celsr1* are found in the *loop tail* and the *crash* mouse, respectively.^5^, ^9^, ^10^ In each, neural tube defects are mild in heterozygous mice, which are viable and fertile, but severe in homozygotes, leading to embryonic lethality.

PCP genes have also been implicated in kidney differentiation, especially controlling tubular diameter, partly through orienting cell divisions.^11^, ^12^, ^13^ We reported that although the initiation of the ureteric bud was normal in homozygous mutant *Vangl2* embryos, subsequent ureteric bud branching within the metanephros was defective.^14^ Furthermore, we^14^ and others^15^ found that these kidneys contained poorly differentiated glomeruli. These defects correlate with reported sites of *Vangl2* mRNA expression, including podocytes and ureteric bud epithelia.^14^, ^16^ Furthermore, knockdown of *Vangl2* in cell culture perturbs the mobility of kidney epithelia,^17^ and *in vivo* the protocadherin *Fat4* genetically interacts with *Vangl2* in kidney cyst formation.^11^

Celsr1 mRNA and protein are localized at diverse sites during metanephrogenesis, including collecting duct stalks, just-formed nephrons, and their proximal tubule derivatives.^14^, ^16^ We therefore hypothesized that *Celsr1* has roles in metanephrogenesis and examined *Celsr1* mutant kidneys. We also studied a possible interaction between *Celsr1* and *Vangl2* in double heterozygous mice. Finally, we asked whether humans born with spina bifida and *CELSR1* mutations might have renal tract malformations.

## Results

### Ureteric branching in the early metanephros of wild-type mice

**Figure S1:**
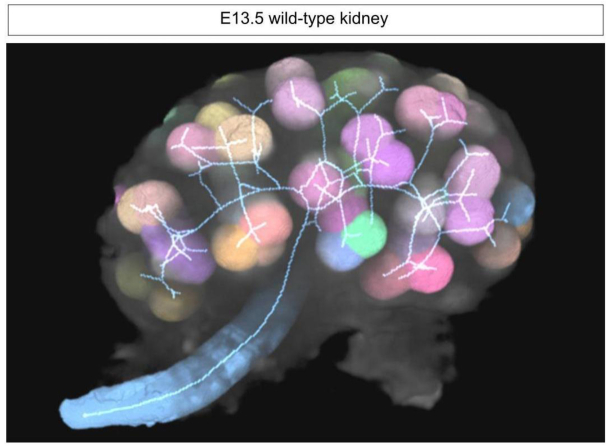
Construction of a 3-dimensional skeletal structure from optical projection tomography images using Tree Surveyor software.

**Figure 1:**
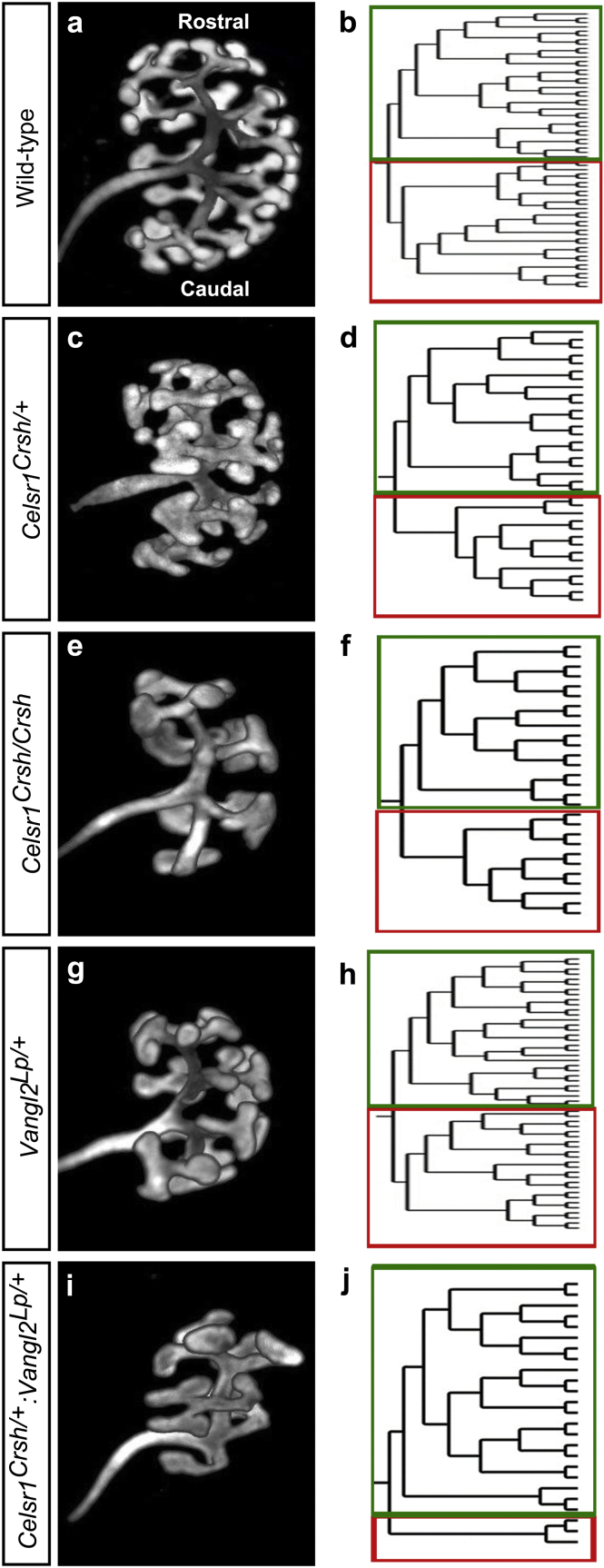
**Assessment of ureteric bud branching in mice with mutations in planar cell polarity genes.** Images of whole metanephroi from E13.5 wild-type (**a**), *Celsr1*^*Crsh/+*^ (**c**), *Celsr1*^*Crsh/Crsh*^ (**e**), *Vangl2*^*Lp/+*^ (**g**), and *Celsr1*^*Crsh/+*^:*Vangl2*^*Lp/+*^ (**i**) mice stained with calbindin-D28K and visualized by optical projection tomography. Branching networks of individual metanephroi from E13.5 wild-type (**b**), *Celsr1*^*Crsh/+*^ (**d**), *Celsr1*^*Crsh/Crsh*^ (**f**), *Vangl2*^*Lp/+*^ (**h**), and *Celsr1*^*Crsh/+*^:*Vangl2*^*Lp/+*^ (**j**) mice. Data are representative of *n =* 3 for each genotype. *Celsr1*, *Cadherin EGF LAG seven-pass G-type receptor 1*; *Vangl2*, *Van Gogh-like 2*.

**Figure 2:**
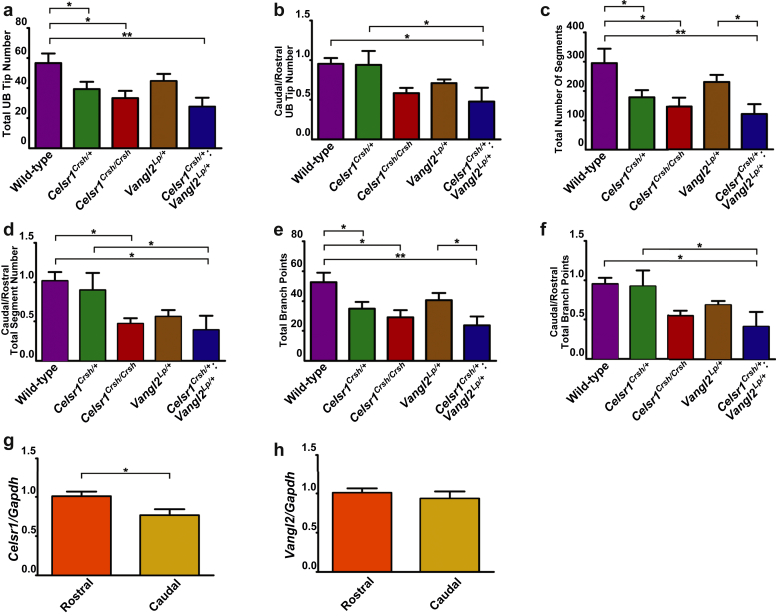
**Quantitative assessment of ureteric bud branching in mice with mutations in planar cell polarity genes.** Quantitative analysis of the total and caudal/rostral ratios of ureteric bud (UB) branch tips (**a,b**), number of “segments” (i.e., sections between branch points plus the tips) in the tree (**c,d**), and number of branch points in the tree (**e,f**) (*n* = 3 for each genotype). mRNA levels of *Celsr1* (**g**) and *Vangl2* (**h**) in the caudal *versus* the rostral compartment of E13.5 wild-type metanephroi (*n* = 6) as assessed by quantitative reverse transcriptase-polymerase chain reaction. Data are presented as mean ± SEM. *P < 0.05 and **P < 0.01 between groups. *Celsr1*, *Cadherin EGF LAG seven-pass G-type receptor 1;* GAPDH, glyceraldehyde-3-phosphate dehydrogenase; *Vangl2*, *Van Gogh-like 2*.

Metanephric kidneys were harvested from wild-type mice at embryonic day (E)13.5 and whole-mount immunostaining for calbindin-D28K was undertaken^18^, ^19^ to visualize the ureteric tree using optical projection tomography (OPT).^20^ Inspection of images of wild-type metanephroi (Figure 1a) revealed a complex ureteric tree. For each kidney, we constructed branching networks using Tree Surveyor software (University of Monash, Melbourne, Australia) (Supplementary Figure S1).^21^ A representative image of the wild-type kidney is shown in Figure 1b. This allowed us to quantify the following: (i) ureteric bud branch tips; (ii) the number of “segments” (i.e., sections between branch points plus the tips); and (iii) the number of branch points. We expressed these results not only for whole organs but also as caudal/rostral ratios. Wild-type metanephroi contained 57 ± 6 ureteric bud branch tips (Figure 2a), equally distributed between caudal and rostral compartments (Figure 2b). Metanephroi contained 295 ± 49 segments (Figure 2c) with similar numbers in caudal and rostral compartments (Figure 2d). There was a total of 53 ± 6 branch points in wild-type metanephroi (Figure 2e) evenly split between caudal and rostral compartments (Figure 2f). Thus, the E13.5 kidney contains a highly branched ureteric tree that is similar in the top and bottom halves of the organ.

### Ureteric branching is simplified in *Celsr1* mutant metanephroi

We then investigated ureteric tree morphogenesis in mice with a point mutation in *Celsr1* (*Celsr1*^*Crsh/Crsh*^). As assessed by looking at OPT images (Figure 1c and e), the *Celsr1*^*Crsh/Crsh*^ ureteric tree appeared smaller and less complex than normal, with heterozygous *Celsr1*^*Crsh/+*^ metanephroi having an intermediate phenotype. Quantification of *Celsr1*^*Crsh/+*^ ureteric tree skeletons (Figure 1d) showed significant reductions in the total number of ureteric bud tips (Figure 2a), branch segments (Figure 2c), and branch points (Figure 2e) compared with wild types (*P* < 0.05 for each parameter). There were no differences in the caudal/rostral ratio in these parameters *versus* wild-type mice. Inspection of OPT images suggested that *Celsr1*^*Crsh/Crsh*^ metanephroi contained more rudimentary ureteric trees *versus* wild-type mice (Figure 1f). This was confirmed by quantification of total ureteric bud tip number (Figure 2a), tree segments (Figure 2c), and branch points (Figure 2e) (*P* < 0.05 for each parameter). The average caudal/rostral ratio for each of these parameters was less than unity, with a significant reduction measured for ureteric tree segments (*P* < 0.05). Thus, the ureteric tree is simplified in *Celsr1* mutant kidneys, with a tendency for a more marked effect in the caudal compartment in homozygous organs.

### *Celsr1* and *Vangl2* interact in ureteric bud growth

Our previous work demonstrated that mice with homozygous missense mutations in *Vangl2* (*Vangl2*^*Lp/Lp*^) also had abnormal ureteric bud branching.^14^ Accordingly, we predicted that there might be an interaction between *Celsr1* and *Vangl2* in kidney development. We tested this by breeding *Celsr1*^*Crsh/+*^ with *Vangl2*^*Lp/+*^ mice. The resulting embryos were produced in the expected Mendelian ratio (Supplementary Table S1). The OPT-imaged ureteric trees of E13.5 *Vangl2*^*Lp/+*^ metanephroi (Figure 1g and h) appeared marginally less complex than wild types. Quantification revealed nonsignificant tendencies for a reduction in branching parameters in the whole organ and caudal/rostral ratios. In contrast, the ureteric tree in *Celsr1*^*Crsh/+*^:*Vangl2*^*Lp/+*^ metanephroi appeared markedly rudimentary (Figure 1i) *versus* wild-type organs. Moreover, the tree appeared asymmetric with less growth in the caudal part of the kidney (Figure 1j). Quantification showed marked growth inhibition in E13.5 metanephroi isolated from *Celsr1*^*Crsh/+*^:*Vangl2*^*Lp/+*^ mice, with significantly lower numbers of total ureteric bud tips (28 ± 6, Figure 2a), ureteric tree segments (121 ± 34, Figure 2c), and branch points (24 ± 6, Figure 2e) compared with wild types (*P* < 0.01 in each parameter). The branching of the caudal region of the kidney in *Celsr1*^*Crsh/+*^:*Vangl2*^*Lp/+*^ mice was especially affected, with significant reductions in the caudal/rostral ratios *versus* wild-type organs with regard to ureteric bud tip number (Figure 2b), segments (Figure 2d), and branch points (Figure 2f) (*P* < 0.05 for all parameters). The results support the hypothesis that these 2 PCP genes are together required for normal ureteric tree morphogenesis, and this is especially apparent in the caudal part of the developing kidney. Given the caudal truncation found in *Celsr1*^*Crsh/Crsh*^ and *Celsr1*^*Crsh/+*^:*Vangl2*^*Lp/+*^ metanephroi, we asked whether the expression of either or both PCP genes differ between rostral and caudal compartments during normal development. We dissected rostral and caudal segments of E13.5 metanephroi and by quantitative reverse transcriptase-polymerase chain reaction found that the level of *Celsr1* transcripts was modestly but significantly less (*P* < 0.05) in the caudal *versus* the rostral compartment (Figure 2g). In contrast, there was no difference for *Vangl2* (Figure 2h).

### Histological analyses of later stage metanephroi

**Figure 3:**
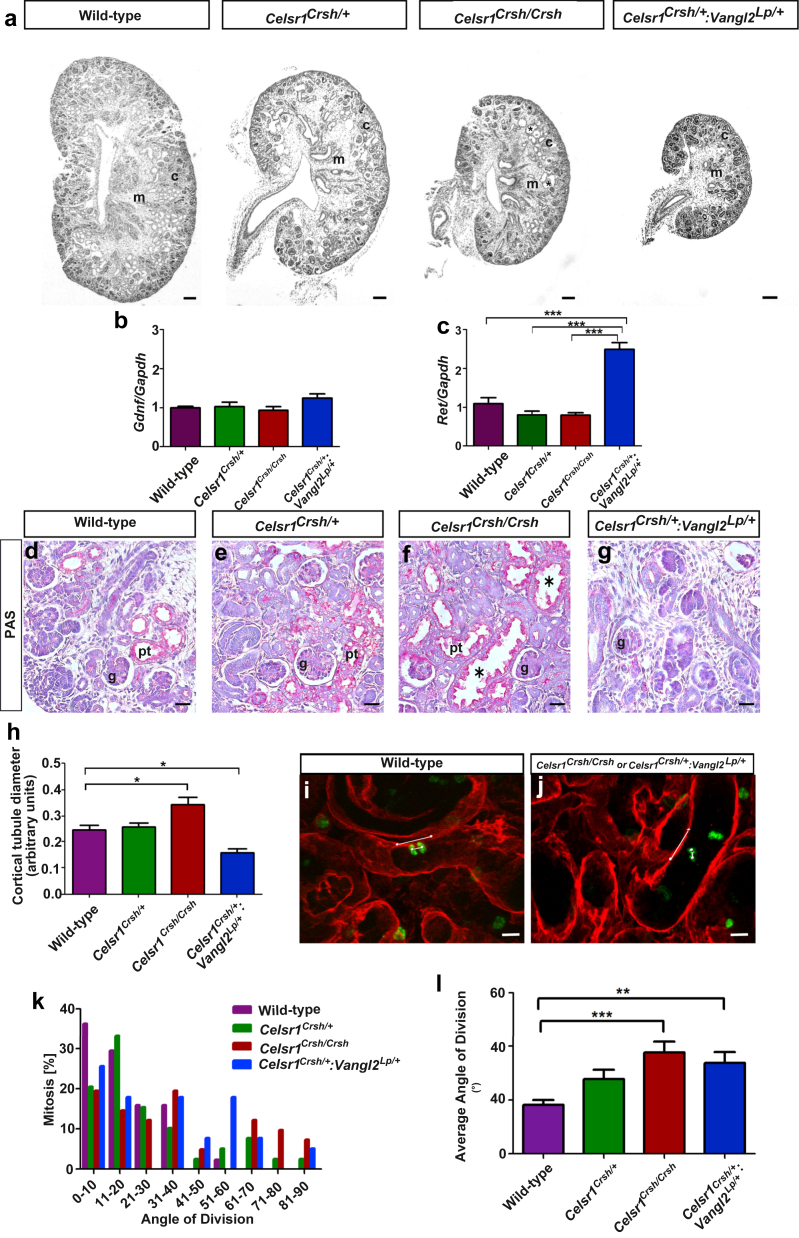
**Histological analysis of E17.5 kidneys in mice with mutations in planar cell polarity genes.** Low-power images of kidneys from E17.5 wild-type, *Celsr1*^*Crsh/+*^, *Celsr1*^*Crsh/Crsh*^, and *Celsr1*^*Crsh/+*^:*Vangl2*^*Lp/+*^ mice (**a**). There was clear demarcation between the cortex (c) and medulla (m) in kidneys from all genotypes except for *Celsr1*^*Crsh/+*^:*Vangl2*^*Lp/+*^ mice. Note the presence of dilated tubules (*) in *Celsr1*^*Crsh/Crsh*^ kidneys. mRNA levels of *Gdnf* (**b**) and *Ret* (**c**) in E17.5 kidneys (*n =* 5–7) as assessed by quantitative reverse transcriptase-polymerase chain reaction. High-power images of the outer cortex of kidneys from wild-type (**d**), *Celsr1*^*Crsh/+*^ (**e**), and *Celsr1*^*Crsh/Crsh*^ (**f**) mice containing glomeruli (**g**) and proximal tubules (pt) with PAS-positive material in their brush borders. Note dilated tubules (∗) in *Celsr1^Crsh/Crsh^* kidneys. *Celsr1*^*Crsh/+*^:*Vangl2*^*Lp/+*^ kidneys (**g**) contained immature glomeruli and rudimentary tubular structures. Measurement of proximal tubular diameter (**h**) in wild-type, *Celsr1*^*Crsh/+*^, *Celsr1*^*Crsh/Crsh*^, and *Celsr1*^*Crsh/+*^:*Vangl2*^*Lp/+*^ mice (*n =* 4–6). Examples of mitotic orientation measurements in tubules from E17.5 kidneys of wild-type (**i**) and *Celsr1*^*Crsh/Crsh*^ mice (**j**). Quantification of mitotic orientation showing % of cells with specific angles of division (**k**) and average angle of division in tubular cells (**l**) (*n =* 3–5). Bar = 125 μm in (**a**), 50 μm in (**d–g**), and 15 μm in (**i–j**). *P < 0.05, **P < 0.01, and ***P < 0.001 between groups. *Celsr1*, *Cadherin EGF LAG seven-pass G-type receptor 1;* GAPDH, glyceraldehyde-3-phosphate dehydrogenase; PAS, periodic acid–Schiff; PCP, planar cell polarity; *Vangl2*, *Van Gogh-like 2*.

Next, we analyzed E17.5 metanephroi. At this stage, although ureteric bud branch tips are still found in the outermost cortex, the bulk of the cortex is filled by nephrons. Loops of Henle emanate from these nephrons and grow into the medulla between maturing collecting ducts. The medulla also begins to form the papilla, which projects into the renal pelvis. Our previous studies showed that E17.5 *Vangl2*^*Lp/Lp*^ kidneys contained immature glomeruli and a hypoplastic medulla, whereas *Vangl2*^*Lp/+*^ organs appeared near normal.^14^ Accordingly, our current E17.5 analyses focused on comparing *Celsr1*^*Crsh/+*^, *Celsr1*^*Crsh/Crsh*^, and *Celsr1*^*Crsh/+*^*:Vangl2*^*Lp/+*^ organs with wild-type kidneys. Inspection of sagittal sections through the midline of organs (Figure 3a) showed an overall growth retardation of *Celsr1*^*Crsh/+*^*:Vangl2*^*Lp/+*^*>Celsr1*^*Crsh/Crsh*^>*Celsr1*^*Crsh/+*^ *versus* wild-type organs. Glial cell line–derived neurotrophic factor (Gdnf) is a paracrine factor secreted by metanephric mesenchyme that induces ureteric tree growth after binding to the Ret receptor tyrosine kinase, expressed in ureteric bud tips.^1^, ^22^ We reasoned that impaired growth of PCP mutant kidneys might be explained by a lack of one or both of these molecules but by quantitative reverse transcriptase-polymerase chain reaction found no depletion of either *Gdnf* or *Ret* in any of the 3 mutant genotypes *versus* wild types. Moreover, in *Celsr1*^*Crsh/+*^:*Vangl2*^*Lp/+*^ kidneys, *Gdnf* was increased 1.25 ± 0.11-fold (*P* = 0.08) and *Ret* transcripts were significantly (*P* < 0.001) increased 2.50 ± 0.17-fold compared with wild-type kidneys (Figure 3b and c).

**Figure S2:**
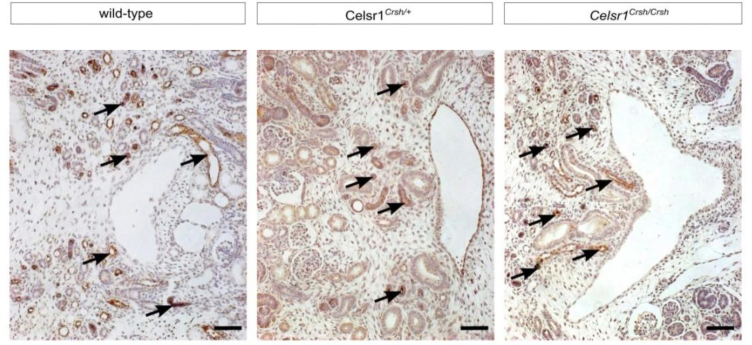
Uromodulin staining in wild-type, *Celsr1*^*Crsh/+*^, and *Celsr1*^*Crsh/Crsh*^ kidneys. Note positive staining in all 3 genotypes (indicated by arrows). Bar = 75 μm, and images are representative of *n =* 4-6 for each genotype.

**Figure S3:**
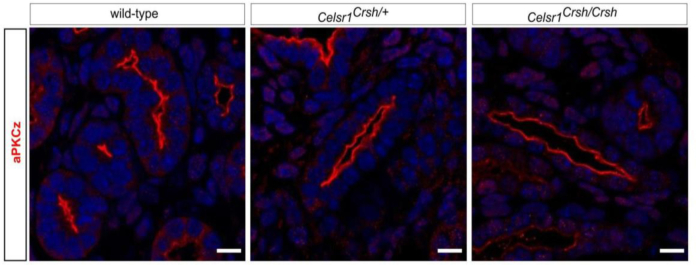
Apical membrane marker atypical protein kinase C (aPKCζ) staining in wild-type, *Celsr1*^*Crsh/+*^, and *Celsr1*^*Crsh/Crsh*^ kidneys. Bar = 50 μm, and images are representative of *n =* 4-6 for each genotype.

We also used histological sections to examine the internal structure of E17.5 kidneys. Wild-type E17.5 metanephroi had a clear demarcation between the cortex and the medulla, and its center contained a slit-like renal pelvis (Figure 3a). The cortex of wild-type kidneys contained glomeruli at different stages of maturation and proximal tubules with differentiated apical brush-borders (Figure 3d). Although somewhat smaller than normal organs, E17.5 heterozygous *Celsr1*^*Crsh/+*^ kidneys had no other overt structural aberrations (Figure 3a and e). In contrast with heterozygotes, *Celsr1*^*Crsh/Crsh*^ kidneys had abnormal histology, with apparently dilated cortical tubules (Figure 3a and f). Despite this aberration, normal-appearing uromodulin-positive loops of Henle^19^ were found in the medulla of *Celsr1*^*Crsh/Crsh*^ kidneys, and the mutant organ contained a renal pelvis (Supplementary Figure S2). Immunohistochemistry for the apical membrane marker atypical protein kinase C^23^ showed no aberration in polarity in *Celsr1*^*Crsh/Crsh*^ kidneys (Supplementary Figure S3). E17.5 *Celsr1*^*Crsh/+*^:*Vangl2*^*Lp/+*^ metanephroi had markedly truncated caudal regions (Figure 3a), and the internal structure of these compound heterozygous organs was abnormal. The cortex contained rudimentary but undilated tubules, and the medulla was hypoplastic, lacking a papilla and renal pelvis (Figure 3a and g).

### Assessment of mitotic orientation in cortical tubules

To confirm our impression of dilated cortical tubules in *Celsr1*^*Crsh/Crsh*^ kidneys, we quantified the diameter, which was similar in wild-type and *Celsr1*^*Crsh/+*^ mice but significantly increased in *Celsr1*^*Crsh/Crsh*^ organs (Figure 3h). In the healthy developing kidney, normal tubule elongation is accompanied by a predominantly proximal/distal orientation of mitotic spindles^24^ and PCP proteins have been implicated in the latter event.^11^, ^12^, ^13^ We hypothesized that the increased tubule diameter in *Celsr1*^*Crsh/Crsh*^ mice might be associated with a randomized orientation of mitotic spindles. Accordingly, E17.5 kidneys were stained with an antiphosphohistone H3 antibody and tubular cells in late anaphase or telophase were identified. The orientation of cell division was determined by measuring the angle between the mitotic spindles of dividing cells and the longitudinal axis of the tubules stained with an anti-entactin antibody. In wild-type mice, 66% of mitotic spindles were orientated within 20° of the longitudinal axis of the tubule (Figure 3i and k), whereas only 34% of spindles were orientated in this manner in *Celsr1*^*Crsh/Crsh*^ mice. Furthermore, in wild-type tubules only 2% of spindles were orientated at an angle of >40° from the axis whereas 34% of spindles in *Celsr1*^*Crsh/Crsh*^ were orientated in this way (Figure 3j and k). Tubular cells in heterozygous *Celsr1*^*Crsh/+*^ kidneys had an intermediate phenotype with 54% and 20% of the mitotic spindles orientated <20° and >40°, respectively (Figure 3k). We calculated the average angle of orientation in at least 40 cells from 3 to 5 kidneys from each genotype (Figure 3l). We found a significant (*P* < 0.001) increase in the angle of orientation between *Celsr1*^*Crsh/Crsh*^ and wild-type kidneys, but no significant difference (*P* = 0.09) between *Celsr1*^*Crsh/+*^ and wild-type kidneys. In *Celsr1*^*Crsh/+*^:*Vangl2*^*Lp/+*^ kidneys, the average diameter of tubule lumens was less than normal (Figure 3h). However, the mitotic orientation in tubule cells was increased in *Celsr1*^*Crsh/+*^:*Vangl2*^*Lp/+*^ kidneys, with 44% and 38% of spindles orientated <20° and >40° of the longitudinal axis of the tubule, respectively (Figure 3k) and a significant (*P* < 0.05) increase in the average angle of orientation compared with wild-type kidneys (Figure 3l).

### Examination of glomeruli in E17.5 metanephroi

**Figure S4:**
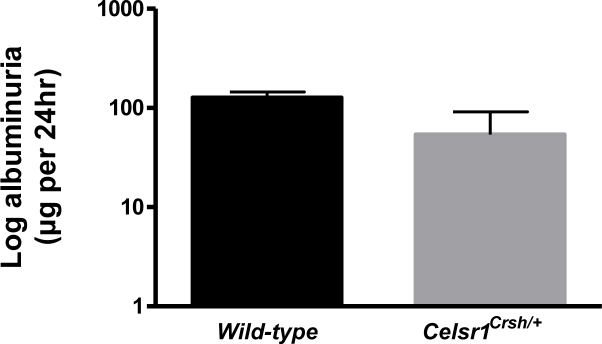
Albumin excretion in 3-month-old wild-type (*n =* 7) and *Celsr1*^*Crsh/+*^ (*n =* 3) mice. There was no significant difference between the 2 groups.

**Figure 4:**
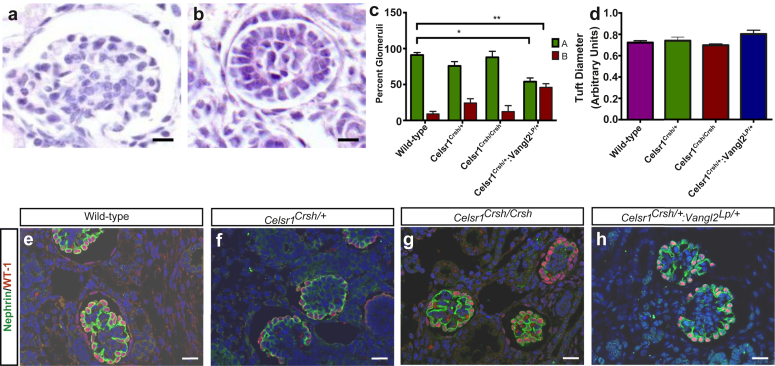
**Assessment of glomeruli of E17.5 kidneys in mice with mutations in planar cell polarity genes.** Representative images illustrating the 2 categories to which glomeruli were assigned: (**a**) Normal mature glomerulus and (**b**) immature glomerulus with <2 capillary loops. Quantification of glomerular morphology (**c**) showed a significant decrease in the proportion of normal glomeruli and an increase in the amount of immature glomeruli in *Celsr1*^*Crsh/+*^:*Vangl2*^*Lp/+*^ compared with wild-type mice. Assessment of tuft diameter (**d**) revealed no differences between any of the genotypes assessed. WT-1 and nephrin double immunostaining showed no difference in the glomerular expression pattern between wild-type, *Celsr1*^*Crsh/+*^, *Celsr1*^*Crsh/Crsh*^, and *Celsr1*^*Crsh/+*^:*Vangl2*^*Lp/+*^mice (**e–h**). Data are representative of *n =* 4–6. Bar = 50 μm in all panels. Data are presented as mean ± SEM. *P < 0.05 and **P < 0.01 between groups. *Celsr1*, *Cadherin EGF LAG seven-pass G-type receptor 1; Vangl2*, *Van Gogh-like 2*; WT-1, Wilms tumor-1.

Our previous work demonstrated that late gestation homozygous *Vangl2*^*Lp/Lp*^ kidneys contain dysmorphic glomeruli.^14^ Because Celsr1 protein is immunolocalized in normal maturing glomeruli, a blinded observer assessed the extent of glomerular maturation in E17.5 wild-type, *Celsr1*^*Crsh/+*^, and *Celsr1*^*Crsh/Crsh*^ mice and *Celsr1*^*Crsh/+*^:*Vangl2*^*Lp/+*^ kidneys. A total of 15 to 20 glomeruli per kidney were scored as either a normal mature glomerulus (Figure 4a) or an immature glomerulus with <2 capillary loops (Figure 4b). Unlike mice with homozygous mutations in *Vangl2*, we found no change in glomerular maturation in *Celsr1*^*Crsh/+*^ or *Celsr1*^*Crsh/Crsh*^ kidneys (Figure 4c**)** or the average diameter of the glomerular tuft (Figure 4d). We immunodetected Wilms tumor-1 and nephrin, molecules expressed in podocytes,^25^ and found no apparent difference in patterns between *Celsr1* mutant and wild-type glomeruli (Figure 4e-g). *Celsr1*^*Crsh/+*^*:Vangl2*^*Lp/+*^ organs contained immature glomeruli *versus* wild types; 46.0% ± 5.1% of glomeruli in the double heterozygotes were immature compared with 9.0% ± 3.5% in wild-type kidneys (*P* < 0.01) (Figure 4c). However, there was no difference in the average diameter of the glomerular tuft between *Celsr1*^*Crsh/+*^*:Vangl2*^*Lp/+*^ and wild-type littermates (Figure 4d), nor any overt changes in immunostaining patterns of Wilms tumor-1 and nephrin (Figure 4h). We also assessed glomerular function by examining albumin excretion in 3-month-old *Celsr1*^*Crsh/+*^ mice and found no difference compared with wild-type littermates (Supplementary Figure S4). It was not possible to assess postnatal kidney function in either *Celsr1*^*Crsh/Crsh*^ or *Celsr1*^*Crsh/+*^:*Vangl2*^*Lp/+*^ mice because they exhibited severe cranioachischisis (at a penetrance of 100%) leading to lethality before birth.^5^, ^10^

### Renal anomalies *and CELSR1* mutations in individuals with spina bifida

In mice, *Celsr1* mutations cause neural tube defects, and the above results show that homozygous mice have severely dysmorphic kidneys. Furthermore, heterozygous *CELSR1* variants have been reported in humans with spina bifida.^26^ Therefore, we asked whether mutations in *CELSR1* may coexist with renal tract anomalies in this group of patients, as assessed by fetal ultrasound scan screening and renal investigations in the immediate postpartum period. Using data from the California Birth Defects Monitoring Program, 13 individuals with spina bifida and heterozygous variants in *CELSR1* were identified (Table 1).^8^ One individual carrying a variant (c.5719_5720 del TG) that generates a truncated CELSR1 protein had unilateral “renal agenesis,” with the opposite renal tract affected by hydronephrosis and hydroureter. Another individual, also carrying a protein-truncating mutation of *CELSR1* (c.5050_5051 ins GT), had bilateral hydronephrosis, and 3 other patients, all carrying missense *CELSR1* variants, were diagnosed as having hydronephrosis, which was bilateral in 2 patients.

## Discussion

Numerous molecules that control the initiation, growth, and differentiation of the metanephros have been identified.^1^, ^2^ These include factors that stimulate ureteric bud initiation and branching, mesenchymal to epithelial transition, and maturation of kidney epithelia. Several of these genes have been found to be mutated in humans born with kidney or lower renal tract malformations or both.^2^, ^27^, ^28^ Despite this, little is known about how the kidney’s rostrocaudal symmetry becomes established. Moreover, to date, only a minor subset of people with renal malformations have been shown to carry defined mutations of genes normally active in kidney development.

Our study shows that *Celsr1* is essential for normal murine kidney growth and differentiation. In early kidney development it is required for ureteric tree growth, whereas later in gestation it prevents tubule overgrowth in association with orientation of mitosis along the longitudinal axis of kidney tubules. We also demonstrated an interaction between *Celsr1* and *Vangl2* in growth of the ureteric tree in the caudal compartment of the developing kidney and that these 2 genes together are required for glomerular maturation. Finally, we demonstrated that humans with *CELSR1* mutations and neural tube defects can have significant renal malformations. These results provide further evidence that PCP gene mutations are important in mammalian kidney development and reveal an unexpected role for PCP molecules in rostrocaudal patterning during organogenesis.

A main finding of our study was the defective ureteric tree branching and reduction in tip numbers observed in *Celsr1*^*Crsh/Crsh*^ and *Celsr1*^*Crsh/+*^:*Vangl2*^*Lp/+*^ kidneys. Branching morphogenesis of the ureteric tree is associated with cell migration^29^ and changes in cell shape.^30^ The latter processes are controlled by cytoskeletal rearrangements regulated by PCP through downstream signaling of Rho GTPase^4^ and so may be perturbed in *Celsr1*^*Crsh/Crsh*^ mice. Our previous studies in *Vangl2*^*Lp/Lp*^ mice^14^ showed irregular actin expression in proximal tubules. Furthermore, embryonic lung cultures from *Celsr1*^*Crsh/Crsh*^ mice have impaired branching, which can be recovered by the addition of a Rho kinase activator.^31^ In our current study, we found no changes in the mRNA levels of actin depolymerising factors (*Cfl1*, *Dstn*, *Tmsb4x*) between the kidneys of wild-type and *Celsr1*^*Crsh/Crsh*^ mice at E17.5 (data not shown), although this cannot rule out changes at earlier time points in kidney development. Celsr1 has also been implicated in the migration of several cell types including branchiomotor neurons,^32^ inflammatory cells,^33^ and endothelial cells.^34^ Future studies could assess cell migration in *Celsr1*^*Crsh/Crsh*^ mice using time-lapse microscopy together with genetic labeling methods to examine ureteric bud branching.^29^

The aberrant ureteric tree phenotype was particularly pronounced in the caudal region of *Celsr1*^*Crsh/+*^:*Vangl2*^*Lp/+*^ kidneys. *Celsr1* and *Vangl2* are expressed in the developing kidney in partially overlapping patterns in the ureteric bud, metanephric mesenchyme, and podocytes.^14^, ^16^ Furthermore, immunoprecipitation studies in human embryonic kidney cells have shown a physical interaction between the encoded proteins.^8^ The branching asymmetry in the caudal region in *Celsr1*^*Crsh/+*^:*Vangl2*^*Lp/+*^ metanephroi could alternatively relate to the shortened body axis in PCP mutants due to impaired convergent extension movements,^35^ placing a physical constraint on metanephric morphogenesis. This could be examined in future experiments by culturing early metanephroi *ex vivo* and assessing ureteric branching by detailed OPT imaging and analyses. Other studies have reported that addition of bone morphogenetic protein 4 to explanted wild-type metanephroi impairs development of the organ, with a more pronounced effect in the caudal part.^36^, ^37^ It has been suggested that this might be mediated by disruption of mesenchymal-derived factors that normally enhance growth of the ureteric tree.^37^ In contrast to this idea, we measured upregulated expression of *Gdnf*, and its *Ret* receptor, in compound mutant kidneys. We also found no significant rostrocaudal asymmetry in wild-type metanephroi harvested at E13.5. However, Cain et al^36^ reported that the caudal section of wild-type metanephroi had a modest but significant deficit in its ureteric tree *versus* the rostral section using E12.5 rudiments maintained in organ culture for 2 days. Furthermore, Short et al^38^ used OPT imaging and Tree Suveyor software in wild-type mice and showed initial lobes within the branching tree with differences in rostral *versus* caudal branching. In this respect, it was notable that we measured a modest depletion in *Celsr1* transcripts in the caudal *versus* the rostral portion of wild-type E13.5 kidneys.

Another finding in our study was that *Celsr1*^*Crsh/Crsh*^ kidneys have a major deviation from normal in orientation of mitotic spindles, which was associated with the presence of shorter and wider cortical tubules. Abnormal mitotic orientation has been reported in *Celsr1* null mice in the epithelial cells of the oviduct^39^ and hair follicles.^40^ It has been proposed that the primary cilium plays a key role in mitotic cell division^41^ and studies using null mice have proposed that *Celsr1* (together with *Vangl2* and *Fzd3*) coordinates the positioning of the cilia and harmonizes the orientation and direction of ciliary beating within neighboring cells.^42^ The effect of the point mutation in *Celsr1*^*Crsh/Crsh*^ mice on the primary cilia has not been established, but it could be postulated that it would lead to a defect in cilia function, which would result in the abnormal mitotic orientation seen in the kidneys from these mice. The almost randomized mitotic spindle orientation is a possible explanation for the presence of abnormally dilated metanephric tubules in *Celsr1*^*Crsh/Crsh*^ mice. Alternatively, the *Celsr1*^*Crsh/Crsh*^ mice may have a primary deficiency in the elongation of the tubular epithelium, which subsequently results in impaired mitotic orientation. Interestingly, *Celsr1*^*Crsh/+*^:*Vangl2*^*Lp/+*^ kidneys also contained tubule cells with similarly aberrant mitotic orientation but without an associated increase in tubular diameter. One way to reconcile this contradiction is that *Celsr1*^*Crsh/+*^:*Vangl2*^*Lp/+*^ kidneys are generally less differentiated than normal, together with the previous observation that mitotic orientation becomes uniform only, and thus a key determinant of tubule diameter, in more mature tubules.^43^

*Celsr1* has been detected together with several other PCP genes in glomerular podocytes,^14^, ^17^ cells that are highly branched and polarized.^44^ Despite this, *Celsr1*^*Crsh/Crsh*^ mice had no apparent abnormalities in glomerular maturation or tuft diameter. Furthermore, we found no difference in albumin excretion between postnatal *Celsr1*^*Crsh/+*^ mice and their wild-type littermates. Most likely, not all PCP genes are critical for glomerular function. Mice engineered to lack the PCP protein Scribble in podocytes have no glomerular abnormalities.^45^ In contrast, both *Vangl2*^*Lp/Lp*^ and podocyte-specific *Vangl2* knockout mice contain smaller and immature glomeruli.^14^, ^15^ It is possible that *Celsr1* may play a role in the setting of glomerular disease. A study showed induction of glomerular mRNA levels of several PCP genes in the early stages of a murine model of nephrotoxic nephritis, although the levels of *Celsr1* were not evaluated.^15^ Furthermore, podocyte-specific deletion of *Vangl2* exaggerated the severity of the nephrotoxic nephritis model with enhanced glomerulosclerosis.^15^ Therefore, future studies may examine the role of *Celsr1* in glomerular disease using heterozygous *Celsr1*^*Crsh/+*^ mice or transgenic animals with glomerular-specific deletion of *Celsr1*.

It has long been noted^46^, ^47^ that a subset of babies with spina bifida has congenital renal tract malformations such as renal agenesis, horseshoe (fused) kidneys, ectopic kidneys, and ureter malformations. These anomalies are distinct from physical disruptions of the shape of the renal tract that can occur in individuals with spina bifida who survive infancy; these acquired defects form gradually and are secondary to functional bladder outflow obstruction. The renal tract malformations that we documented in 5 of 13 babies with spina bifida carrying *CELSR1* variants represent true developmental defects because they were detected in the fetal or immediate postnatal periods. In a European renal anomaly detection program,^48^ it was reported that unilateral renal agenesis occurred in 56 of 709,030 live births, stillbirths, and induced abortions in the general population. In contrast, in our cohort of 13 individuals with spina bifida and *CELSR1* variants, we recorded 1 case of unilateral renal agenesis, a significant difference (*P* = 0.001, Fisher exact test) *versus* the control population. In a New Zealand population screening study^49^ of 3856 fetuses having ultrasonography in the last trimester, 298 had hydronephrosis, and 82 of these had persistent hydronephrosis in the first 6 weeks of postnatal life. Of the 13 patients in our spina bifida/*CELSR1* cohort, 5 had hydronephrosis as assessed by radiology in the fetal or early postnatal periods or both. We do not have the clinical information to determine whether these appearances would have been persistent or transient, but the incidence is significantly higher in our cohort than in controls when either considering total cases (*P* = 0.002, Fisher exact test) or only persistent cases (*P* = 0.0001) in the general population. Furthermore, clinical renal scanning results we had at our disposal reported only major malformations, and more subtle anomalies, such as small kidneys, may not have been captured in the reports. At the least, our current results should encourage further studies to determine whether spina bifida and PCP mutations (including *CELSR1* and *VANGL2*) coexist with kidney malformations.

Given that the main renal tract malformation we detected in our patient cohort was hydronephrosis, how might this relate to our observations in mutant mice? Here, some caution is required in directly comparing the human and mouse results because the limit of our fetal mouse study was E17.5, a stage when the definitive pelvis is yet to form, a process in part driven by papillary growth and remodeling of early branches of the ureteric bud.^38^ With this caveat in mind, it is of note that the medulla of E17.5 *Celsr1*^*Crsh/+*^ and *Celsr1*^*Crsh/Crsh*^ fetuses appeared simplified compared with wild-type littermates (compare Figure 3b-c with a). Moreover, according to GenitoUrinary Development Molecular Anatomy Project,^16^ Celsr1 is expressed not only in the kidney itself but also in smooth muscle of the mature ureter, and primary defects in myogenic differentiation can lead to functional defects in peristalsis that manifest as hydronephrosis.^50^ Therefore, in future, detailed ascertainment of medullary branch patterns and ureteric differentiation in PCP mutant mice may be informative.

## Materials and Methods

### Animal models

All animal procedures were approved by the UK Home Office. *Celsr1*^*Crsh/+*^ heterozygous mice^31^ were bred to generate *Celsr1*^*+/+*^, *Celsr1*^*Crsh/+*^, and *Celsr1*^*Crsh/Crsh*^ littermates. In some experiments, *Celsr1*^*Crsh/+*^ were bred with *Vangl2*^*Lp/+*^ mice^14^ to generate wild-type, single and double heterozygotes. All mice were on a C3H/HeH background and their genotype was identified by Sanger sequencing. To assess gene levels in rostral and caudal segments, RNA from these compartments was extracted using the E13.5 right kidneys of CD1 mice.

### OPT imaging

E13.5 kidneys (*n* = 3 for each genotype) were fixed in methanol, permeabilized in 0.2% Triton-X100 in phosphate-buffered saline, and incubated with anticalbindin-D28K (Sigma Aldrich, St. Louis, MO), followed by incubation with appropriate Alexa Fluor 488 or 594 (Thermo Fisher Scientific, Waltham, MA) secondary antibodies.^18^ Samples were counterstained with Hoechst 33342 (Thermo Fisher Scientific), embedded in 1% low melting point agarose, dehydrated in methanol, and cleared in a 1:2 mixture of benzyl alcohol and benzyl benzoate. Images were acquired at resolutions appropriate for the size of the organ by an OPT scanner (Bioptonics, Edinburgh, UK) and data sets reconstructed using nRecon (Bruker microCT, Kontich, Belgium). Tree Surveyor^21^ was used to create branching networks to measure the following: (i) ureteric bud number; (ii) the number of “segments” (i.e., sections between branch points plus the tips); and (iii) the number of branch points. We expressed these results for whole organs and caudal/rostral ratios; in the latter analyses, the first intrarenal branch of the ureteric bud defined the rostral and caudal sections of the tree.

### Histology and immunohistochemistry

Kidneys were fixed in 4% paraformaldehyde, embedded in paraffin, and 5-μm sections cut. In some experiments, periodic acid–Schiff reagent was used. To assess glomerular maturation, a blinded observer assigned 15 to 20 glomeruli per kidney (*n =* 4-6 kidneys for each genotype) to the following categories: (i) normal mature glomerulus and (ii) immature glomerulus with <2 capillary loops. The proportion of glomeruli in each category for every kidney was determined. The average diameter was assessed in at least 20 cortical tubules in each E17.5 kidney (*n =* 4-6 kidneys for each genotype). Immunohistochemistry was performed as described,^51^ with sections incubated with 1 or more of the following antibodies: anti–atypical protein kinase C (Santa Cruz Biotechnology, Dallas, TX), anti-nephrin (Progen Biotechnik, Heidelberg, Germany), antiuromodulin (Santa Cruz Biotechnology), and anti–Wilms tumor-1 (Acris Antibodies, Herford, Germany). Sections were incubated with appropriate horseradish peroxidase or AlexaFluor 488/594/633 secondary antibodies. Negative controls consisted of omission of primary antibodies.

### Measurement of mitotic orientation

Cryosections (30 μm) from E17.5 kidneys were double-stained with anti–phosphohistone H3 (Sigma) and anti-entactin antibodies (LifeSpan Biosciences, Seattle, WA). Sections were imaged by confocal microscopy and z-stacks were reconstructed in Imaris (Bitplane AG, Zurich, Switzerland). The orientation of cell division was determined as previously described.^52^ A minimum of 40 cells were assessed in 3 to 5 kidneys for each genotype.

### Real-time polymerase chain reaction

RNA was extracted and 500 ng used to prepare cDNA. Quantitative reverse transcriptase-polymerase chain reaction was performed for *Celsr1*, *Gdnf, Ret*, and *Vangl2* on a CFX96 Real-Time PCR System (Bio-Rad Laboratories, Hemel Hempstead, UK) using SsoAdvanced Supermix (Bio-Rad Laboratories) with *Gapdh* as a house-keeping gene. Fold-changes in gene expression were determined by using the 2^−ΔΔ*C*T^ method and expressed relative to levels detected in wild-type mice. Primer details available on request.

### Assessment of albuminuria

Urine was collected from 3-month-old *Celsr1*^*Crsh/+*^ and wild-type littermates by housing them individually in metabolic cages. Albumin concentrations were measured by enzyme-linked immunosorbent assay (Bethyl Laboratories, Montgomery, Alabama).^53^

### Human subjects

Data were obtained from a population-based case-control study conducted by the California Birth Defects Monitoring Program.^54^ To identify mutations, the *CELSR1* gene (NM_014246) was sequenced in 192 infants with isolated spina bifida without other major birth defects (cases) and 190 nonmalformed infants (controls).^8^ Renal tract anomalies were assessed from fetal ultrasound scan screening and investigations in the immediate postpartum period.

### Statistical analyses

Data were presented as means ± SEM. Differences between 2 groups were evaluated by Mann-Whitney *U* test. Three or more groups were assessed by 1-way analysis of variance with least square difference *post hoc* test. Significance was accepted at *P* < 0.05.

## Disclosure

All the authors declared no competing interests.

## Figures and Tables

**Table 1:** Renal tract defects in 5 of 13 individuals with spina bifida and heterozygous variants in *CELSR1*

Nucleotide change	Amino acid change	Genitourinary defects
c.3068C.G	p.Ala1023Gly	No renal anomaly
c.3372C.G	p.Ile1124Met	No renal anomaly
c.4085C.T	p.Thr1362Met	No renal anomaly
c.4228G.A	p.Gly1410Arg	No renal anomaly
c.4927C.T	p.Arg1643Trp	Unilateral hydronephrosis
c.5050_5051 ins GT	Predicted truncated protein	Bilateral hydronephrosis, severe on left
c.5461G.T	p.Val1821Leu	Bilateral hydronephrosis
c.5473G.A	p.Gly1825Ser	No renal anomaly
c.5719_5720 del TG	Predicted truncated protein	Undetectable left kidney, with hydronephrosis and hydroureter of the right renal tract
c.6184G.A	p.Gly2062Ser	Bilateral hydronephrosis
c.7060C.T	p.Arg2354Cys	No renal anomaly
c.7489C.T	p.Arg2497Cys	No renal anomaly
c.8632G.A	p.Gly2878Ser	No renal anomaly

*CELSR1*, *Cadherin EGF LAG seven-pass G-type receptor 1.*

## Supplementary Material

Table S1 Genotype outcomes following the breeding of *Celsr1*^*Crsh/+*^ with *Vangl2*^*Lp/+*^ mice, both on the same C3H/HeH background. The resulting E13.5 embryos were produced in the expected Mendelian ratio
