# Supplementary material for: Planar cell polarity genes Celsr1 and Vangl2 are necessary for kidney growth, differentiation, and rostrocaudal patterning
Source: Kidney Int. 2016 Dec;90(6):1274–84. doi: 10.1016/j.kint.2016.07.011 (PMC5126096; doi:10.1016/j.kint.2016.07.011)
Supplement: Table S1 — Genotype outcomes following the breeding of Celsr1Crsh/+ with Vangl2Lp/+ mice, both on the same C3H/HeH background. The resulting E13.5 embryos were produced in the expected Mendelian ratio [file mmc1.docx]

| **Genotype** | **wild-type** | ***Celsr1^Crsh/+^*** | ***Vangl2^Lp/+^*** | ***Celsr1^Crsh+:^Vangl2^Lp/+^*** |
| --- | --- | --- | --- | --- |
| **Number of embryos [n]** | 9 | 9 | 9 | 10 |
| **Expected Mendelian ratios [%]** | 25 | 25 | 25 | 25 |
| **Actual Mendelian ratios [%]** | 24 | 24 | 24 | 27 |

**Supplementary Table 1.**

Genotype outcomes following the breeding of *Celsr1^Crsh/+^* with *Vangl2^Lp/+^* mice, both on the same C3H/HeH background. The resulting E13.5 embryos were produced in the expected Mendelian ratio
